# Influence of load partial factors adjustment on reliability design of RC frame structures in China

**DOI:** 10.1038/s41598-023-34241-5

**Published:** 2023-05-04

**Authors:** Kaikai Cheng, Guangyuan Weng, Zhengjie Cheng

**Affiliations:** 1 Department of Civil Engineering, Xi’an Shiyou University, Xi’an, 710065 China; 2 Department of Civil Engineering, Xi’an University of Architecture and Technology, Xi’an, 710055 China

**Keywords:** Civil engineering, Structural materials

## Abstract

The partial factor method has been widely used in building design and the partial factors to ensure the safety of structures are specified in the adopted codes. The load partial factors in the design expressions have been increased in the lasted code in China, which leads to theoretically increment in reliability and a growth in the consumption of construction materials. However, the influence of load partial factors adjustment on design of building structures arises different points among scholars. Some believe that it has a great impact on the design, some think the influence is small. This makes designers have doubts in the safety of structures and investors are also confused about the cost. In order to illustrate the influence of load partial factor adjustment on safety level and material consumption of RC (Reinforced Concrete) frame structures, reliability analysis and material consumption analysis are performed using First Order Reliability Method (FORM). The approach is carried out according to the load partial factors in Chinese codes of (GB50153-2008) and (GB50068-2018), respectively. Then, the influence of load partial factors adjustment is demonstrated with a case design of RC frame structures with different load partial factors in codes. The results show that the partial factor has a noticeable influence on the reliability index. The adjustment of load partial factors in design leads to an increase of the reliability index, which is about 8–16%. The increase of material consumption used in RC structures is about 0.75–6.29%. And the case indicated that the adjustment of load partial factors mainly result in the increase of reinforcement consumption, while have little effect on the concrete consumption. This study provides an analytical and conclusive insight into the influence of load partial factor adjustment on safety level and material consumption, which is can be applied to a wide range of structures.

## Introduction

Reliability theory has been taken into account for safety evaluation and design purposes in recent decades because of structural uncertainties caused by design and construction stages^1^. The reliability of the structure is fundamentally defined as its ability to comply in the defined lifetime with design criteria^2^. Reliability-based structural analysis of buildings and constructions made from conventional materials such as masonry, steel or concrete is commonly available in literature. The theoretical background and basic principles for the reliability analysis and calculations can be found in Refs.^3–9^, which has been applied in several studies^10–16^.

Designing structures involves ensuring an adequate level of safety. Structural design codes and specifications include rules and guidelines that specify minimum acceptable level of safety. They aim at protecting public health, safety and the general welfare in relation to construction^17^. In practical engineering, reliability and material consumptions are a pair of contradictory factors. Design is to achieve a balance between the safety and economy of structural components, which is represented by the target reliability. The target reliability index should be determined according to the importance of structure, failure consequence, economic index and other factors. The partial factor method has been widely used in design which ensures and reflects the reliability of structural components. This is done by applying the appropriate set of partial factor for resistance and loads and the corresponding design parameters of design expressions in accordance with the recommendations of the codes^18–21^. Based on reliability theory, Honjo et al.^22^ proposed a method to determine the partial factors in the design format for a vertically loaded pile. Biagi et al.^23^ highlighted that the values of the partial safety factors are associated with the distribution types and statistical parameters of variables. Pacheco et al.^24^ obtained the resistance format and the values of partial factors based on explicit reliability analyses of pultruded FRP. Arrayago et al.^25^ presented a set of simplified equations to estimate reliability indices, resistance factors and partial safety factors. Based on the reliability theory and some design restrictions, the optimum load partial factors with large variable load effect ratio are obtained through numerical calculation by Jiang et al.^26^. According to the method of actions combination based on stomachic processes and JC method for computing reliability index, the reference design values and partial factors of basic variables for structural safety and serviceability design are studied in Refs.^27,28^. Considering the difference from building structures, He et al.^29^ analyzed the values of partial coefficient for loads in combination of dead loads and wind loads for plastic greenhouses. According to the reliability analysis of structural members based on the target reliability index, design reference period and statistical parameters of load, Zhang et al.^30^ presented the design expression of the load partial factors under the design reference period of 60 years.

The structural reliability design standards in China mainly include (GBJ 68-84)^31^ and (GB 50153-92)^32^, which have been revised into (GB 50068-2001)^33^ and (GB 50153-2008)^34^. With the development of economic construction, higher requirements for the reliability and safety of building structures are needed in designing structures. The load partial factors in the design expressions have been improved in the lasted code (GB 50068-2018)^35^, and the reliability of building structures has also been improved to a certain extent. The improvement of reliability will certainly increase the consumption of materials, leading to increase the cost of the structure. However, the influence of load partial factors adjustment on design of building structures arises different points among scholars. Some believe that it has a great impact on the design, some think the influence is small. This makes designers have doubts in designing. In order to obtain a more economical and reasonable reliability level, it is necessary to study the reliability indexes and the consumption of material of building structures designed according to the load partial factor in China’s old code (GB50153-2008)^34^ and new code (GB50068-2018)^35^. Lu et al.^36^ discussed the influence of the seismic action adjustments on the seismic design and safety of RC frames. Wu et al.^37^ illustrated the influence of load partial factor adjustment on safety level and material consumption of the masonry structures. Zhang^38^ analyzed the influence of partial coefficient adjustment on the design of shear wall structure. By collecting the distribution types and statistical parameters of different types of light steel members and considering the load effect ratio of light steel structures, Cheng et al.^39^ clarified the changes of the reliability and material consumption of light steel structures before and after the adjustment of the load partial coefficient. As one of the most common structural forms in civil engineering, RC frame structures have been widely used in office, dwelling, school buildings and so on. The study is intended to illustrate the influence of load partial factor adjustment on safety level and material consumption of RC frame structures, reliability analysis and material consumption analysis are performed using FORM. The results can provide reference for the design of RC frame structures.

## Partial factors and related statistical parameters

### Partial factors for loads

The combination of loads controlled by the dead load has been canceled in Chinese structural reliability standards (GB50068-2018)^35^ and then the partial factor for dead loads changes from ${\upgamma }_{{\text{G}}} { = 1}{\text{.2}}$ to ${\upgamma }_{{\text{G}}} { = 1}{\text{.3}}$, the value for variable loads changes from ${\upgamma }_{{\text{G}}} { = 1}{\text{.4}}$ to ${\upgamma }_{{\text{G}}} { = 1}{\text{.5}}$. Therefore, there is only one basic combination of load effect (${1}{\text{.3S}}_{{\text{G}}} { + 1}{\text{.5S}}_{{\text{Q}}}$) in current design. The partial factors for loads adopted in national and international documents and their respective codes are listed in Table 1.

**Table 1:** Partial factors for loads in national and international documents.

Country	Codes	Partial factor for dead load	Partial factor for variable load
China codes (before adjustment)	(GB 50153-2008)^34^	1.2 (1.35)	1.4
China codes (after adjustment)	(GB 50068-2018)^35^	1.3	1.5
American codes	(ACI 318-14)^40^	1.2 (1.4)	1.6
UK codes	(BS 8110:1997)^41^	1.4	1.6
Europe codes	(EN 1990: 2002)^18^	1.35	1.5
International codes (Austria, Italy, Holland, Spain, etc.)	(ISO 2394:2015)^19^	1.4	1.6

### Statistical parameters for loads

Loads shall be classified by their variation in time as dead load and variable load^3,4,42,43^. The dead load of structure mainly refers to its self-weight, which is assumed not change with time in general. Then dead load is modeled as a normally distributed variable^44^ with its coefficient of mean value equal to 1.06 and the coefficient of variation (c.o.v) equal to 0.07.

For the variable load, its probability distribution of the maximum load in the design working life which is given by

1 $${\text{F}}_{{\text{T}}} \left( {\text{x}} \right){ = }\left[ {{\text{F}}\left( {\text{x}} \right)} \right]^{{\text{T}}} ,$$

where ${\text{T}}$ is the design working life of structural members, 50 years is usually adopted. ${\text{F(x)}}$ is the cumulative distribution function for the annual maximum variable loads. ${\text{F}}_{{\text{T}}} {\text{(x)}}$ is the cumulative distribution function of the maximum variable loads in the design working life.

It is widely accepted that the statistical distribution for the variable load is the extreme type and its distribution function is^5,45^

2 $${\text{F}}\left( {\text{x}} \right) = \exp \left\{ { - \exp \left[ { - \alpha \left( {{\text{x}} - u} \right)} \right]} \right\},$$

where $\alpha$($\alpha > 0$) is the scale parameter of the distribution and $u$($- \infty < u < + \infty$) is the location parameter of the distribution. The mean value and standard deviation of the variable load are:

3 $$\mu_{{\text{X}}} = u + \frac{{\text{C}}}{\alpha } \approx u + \frac{0.5772}{\alpha },$$

4 $$\sigma_{{\text{X}}} = \frac{{\uppi }}{\sqrt 6 \alpha } \approx \frac{1.2826}{\alpha },$$

where ${\text{C}}$ is the Euler’s constant.

The distribution types and statistical parameters of permanent action and variable actions are listed in Table 2^46,47^.

**Table 2:** Distribution types and statistical parameters for loads.

Load type	Distribution type	Statistical parameter	
		Coefficient of mean value $\chi_{{\text{S}}}$	Variable coefficient $\delta_{{\text{S}}}$
Permanent load	Normal distribution	1.06	0.07
Floor live load (office)	Extreme valueItype distribution	0.524	0.288
Floor live load (dwelling)	Extreme valueItype distribution	0.644	0.233
Wind load	Extreme valueItype distribution	1.015	0.194
Snow load	Extreme valueItype distribution	1.025	0.225

### Statistical parameters and partial factors for resistance of RC structural members

The resistance of the structure is influenced by many factors. In the case of design, it should be considered that the factors should include the uncertainty in the material properties, geometrical properties and modeling uncertainty^19,20^. The statistical parameters of different structural members are different under different stress states. According to Refs.^20,48^, the distribution of the resistance of the structure can be modeled as a lognormally distributed variable^13,49^. Due to the complexity and nonlinearity of the resistance, an estimation of the probabilistic distribution and the corresponding statistical properties of the resistance model are especially helpful in the reliability analysis. Researchers have carried out the theoretical and experimental investigations on this topic in order to obtain the probabilistic modeling of the properties of elements in China. The statistical parameters for resistance of RC structural members under different stress statuses are given in Table 3^50^.

**Table 3:** Statistical parameters and partial factors for resistance of RC structural members under different stress statuses.

Structural member type	Distribution type	Stress status	Coefficient of mean value $\chi_{R}$	Variable coefficient $\delta_{R}$	Partial factors for resistance $\gamma_{R}$
Reinforced concrete	Lognormal distribution	Axial tension	1.10	0.10	1.18
	Lognormal distribution	Axial compression	1.47	0.17	1.06
	Lognormal distribution	Large eccentric compression	1.16	0.13	1.21
	Lognormal distribution	Bending	1.24	0.10	1.05
	Lognormal distribution	Shear	1.36	0.19	1.21

According to FORM, the design value method of the resistance can then be calculated as:

5 $$R_{{\text{d}}} = {\text{F}}_{{\text{R}}}^{{ - 1}} \left[ {\Phi \left( { - \alpha \beta } \right)} \right],$$

where $R_{{\text{d}}}$ is the design value of the resistance, $\beta$ is the target reliability index, the recommended values are shown in Table 4, $\Phi ( \cdot )$ is the cumulative density function of the standard normal distribution, $\alpha$ is the value of the FORM sensitivity factor and for resistance, it may be taken as 0.8. Based on the design value method and the current design expression, for resistance obeys to lognormal distribution^49^, the partial factor for resistance can be expressed as

6 $${\upgamma }_{{\text{R}}} { = }\frac{{{\upmu }_{{\text{R}}} }}{{{\upchi }_{{\text{R}}} {\text{F}}_{{\text{R}}}^{{ - 1}} \left[ {\Phi \left( { - \alpha \beta } \right)} \right]}} = \frac{{\sqrt {1 + \delta_{{\text{R}}}^{{2}} } }}{{{\upchi }_{{\text{R}}} }}\exp \left\{ { - \alpha \beta \sqrt {\ln \left( {1 + {\updelta }_{{\text{R}}}^{{2}} } \right)} } \right\},$$

where ${\upmu }_{{\text{R}}}$, ${\upchi }_{{\text{R}}}$ and ${\updelta }_{{\text{R}}}$ are the mean value, the coefficient of mean value and the variable coefficient of the resistance respectively. The calculated partial factors for resistance are shown in Table 3.

**Table 4:** Target values for reliability indexes.

	Safety categories		
	I	II	III
Ductile failure	3.7	3.2	2.7
Brittle failure	4.2	3.7	3.2

## Reliability analysis of RC structural members

Within the partial safety factor approach the safety and serviceability of a structure or component is validated by comparing the design values for the action $S_{{\text{d}}}$ and the resistance $R_{{\text{d}}}$:

7 $${\upgamma }_{{0}} \cdot {\text{S}}_{{\text{d}}} \le {\text{R}}_{{\text{d}}} ,$$

where ${\upgamma }_{0}$ is the structural importance coefficient, when the design working life is 50 years, it may be taken as 1.0.

Consider a generic structural member subjected to one permanent load and one live load, the limit state function can be rewritten as follows^14^:

8 $$Z\left( {\text{R,G,Q}} \right) = {\text{R}} - \left( {{\text{G}} + {\text{Q}}} \right),$$

where ${\text{R}}$ is structural resistance, determined by structural geometric parameters (e.g. the cross-section area) and the strength of the material, ${\text{G}}$ is the load effect due to permanent load on structural members; ${\text{Q}}$ is the load effect due to live load on structural members.

For the ultimate limit states, the design value of action effect and the required characteristic value of structural resistance can be expressed as

9 $${\text{S}}_{{\text{d}}} { = }\gamma_{{\text{G}}} {\text{G}}_{{\text{k}}} { + }\gamma_{{\text{Q}}} \gamma_{{\text{L}}} {\text{Q}}_{{\text{k}}} {,}$$

10 $$R_{{\text{k}}} = {\upgamma }_{{\text{R}}} {\upgamma }_{0} S_{{\text{d}}} ,$$

where ${\text{R}}_{{\text{k}}}$, ${\text{G}}_{{\text{k}}}$ and ${\text{Q}}_{{\text{k}}}$ are the characteristic values of the resistance, the effect of permanent load and the effect of live load respectively; ${\upgamma }_{{\text{G}}}$, ${\upgamma }_{{\text{Q}}}$ and ${\upgamma }_{{\text{R}}}$ are the partial factors of the permanent load, the live load and the resistance, respectively; ${\upgamma }_{{\text{L}}}$ is the variable load adjustment factor for the design working life of live loads, when the design working life is 50 years, the value may be taken as 1.0.

**Figure 1:**
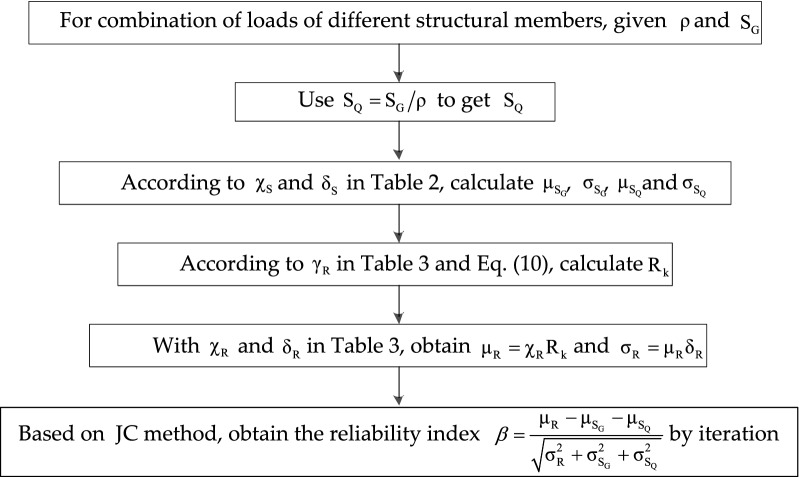
The flowchart for the calculation of the reliability index of the structural members.

In order to consider a wide range of load combinations, a ratio of live load effect to dead load effect $\rho = {\text{Q}}_{{\text{k}}} /{\text{G}}_{{\text{k}}}$ is defined. Since the characteristic value of dead load depends on various factors, the load ratio $\rho$, assumed at the range of (0.1, 2.0) in this paper, is used to determine the reliability indexes^14,51^. For a given load ratio, the characteristic permanent and live loads can be obtained for a given design resistance. With the distribution of basic variables ${\text{R}}$, ${\text{G}}$ and ${\text{Q}}$, reliability analysis of RC structural members is performed by First Order Reliability Method. By making use of this variance reduction approach, the structural reliability analysis is performed in a more computationally efficient way. The flowchart for the calculation of the reliability index of the structural members is shown in Fig. 1. Non-normal variables are treated in accordance to the algorithm developed in Ref.^52^.

**Figure 2:**
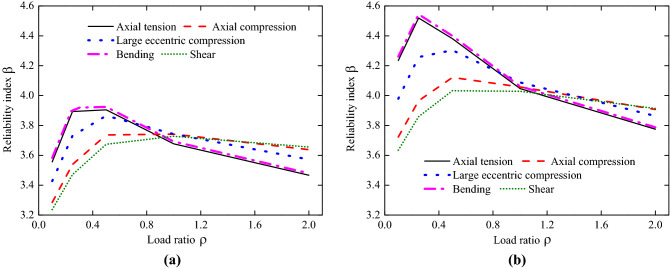
The reliability index for different stress conditions of RC structural members. (**a**) according to partial factors in GB50153-2008; (**b**) according to partial factors in GB50068-2018.

**Figure 3:**
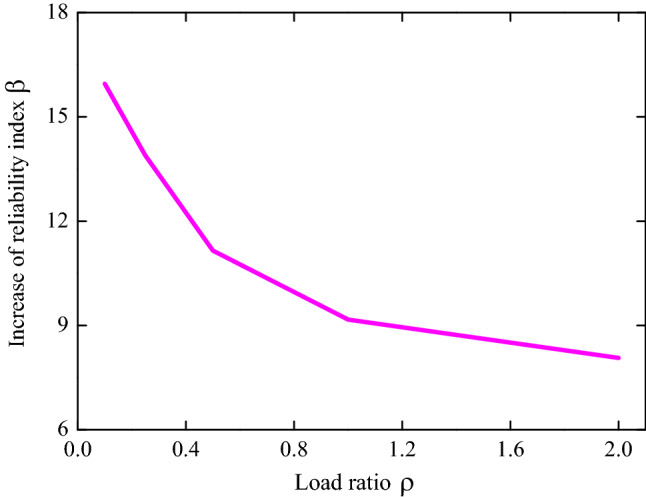
The increase of reliability index due to the adjustment of load partial factors.

The reliability index values of RC structural members are carried out according to the load partial factor in China’s old code (GB50153-2008)^34^ and new code (GB50068-2018)^35^, as shown in Table 5. The variation trend of reliability indexes for different stress conditions of RC structural members with respect to the load ratio are given in Fig. 2. Note that each curve in Fig. 2 consists of four different combinations of actions: (1) permanent load + floor live load (office); (2) permanent load + floor live load (dwelling); (3) permanent load + wind load; (4) permanent load + snow load. First it is found that the curve of bending corresponds to highest values while the curve of shear corresponds to lowest values, which is consistent to the engineering experience. In addition, the values of the reliability index first increase and then decrease with increasing value of the load ratio $\rho$. This is because higher values of the load ratio are associated with more variation of the action effects. Again, it is observed that the partial factor has a noticeable influence on the reliability index. When the values of the partial factor are taken from the new code (GB50068-2018), the reliability index values for different stress conditions of RC structural members increased, and the increased trend and range are basically consistent. That is to say, the adjustment of load partial factors improved the reliability design of RC frame structures. To quantify the influence of the partial factor to the reliability index of RC frame structures, the reliability indexes shown in Fig. 2 are analyzed. The increase of the reliability index due to the adjustment of load partial factors is defined as $\left[ {\beta_{2} - \beta_{1} } \right]/\beta_{1}$, shown in Fig. 3. It can be observed that the increase of the reliability index is about 8–16%.

**Table 5:** Reliability index of RC structural members according to partial factors in different China’s codes.

$\rho$	Axial tension		Axial compression		Large eccentric compression		Bending		Shear	
	Code^34^	Code^35^	Code^34^	Code^35^	Code^34^	Code^35^	Code^34^	Code^35^	Code^34^	Code^35^
0.1	3.556	4.235	3.284	3.723	3.428	3.979	3.583	4.262	3.237	3.634
0.25	3.893	4.520	3.539	3.965	3.730	4.258	3.918	4.544	3.471	3.859
0.5	3.905	4.381	3.736	4.121	3.865	4.304	3.924	4.400	3.674	4.033
1	3.677	4.047	3.742	4.058	3.742	4.088	3.692	4.062	3.728	4.029
2	3.467	3.775	3.638	3.907	3.572	3.863	3.480	3.788	3.656	3.914

## Material consumption analysis of RC structural members

**Figure 4:**
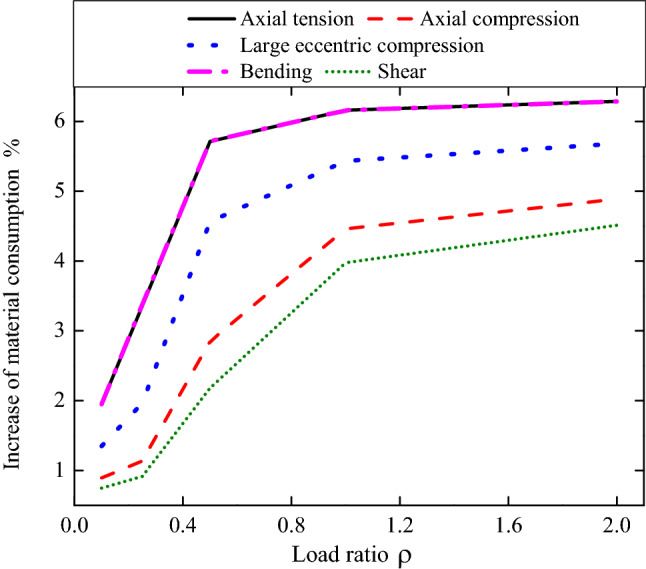
The increase of material consumption due to the adjustment of load partial factors.

The material consumption of structural components is closely related to the target reliability index in codes. The improvement of reliability results in the increase of material consumption. The reliability index and the material consumption are a contradiction between economy and safety. The purpose of design is to find a balance between them. As mentioned in Section “Reliability analysis of RC structural members”, the reliability index values increased with the adjustment of load partial factors, so did the material consumption used in RC structures. For the sake of completeness, the material consumption analysis of RC structural members is also investigated. The materials used in structural components are determined by the load effects to be resisted, i.e. the resistance of the components. According to the calculation process of the reliability index proposed in Section “Reliability analysis of RC structural members”, the material consumption of structural component can be measured by their resistance evaluated at the design points indirectly. The material consumption analysis of RC structural members are carried out according to the load partial factor in China’s old code (GB50153-2008)^34^ and new code (GB50068-2018)^35^, as shown in Table 6. The increase of material consumption due to the adjustment of load partial factors is given in Fig. 4. The results show that the material consumption increases with the increasing of load ratio under all stress statuses and the increase of the material consumption is about 0.75–6.29%. Under different stress statuses, the increase of material consumption of shear is the smallest, followed by axial compression, and the largest is the bending.

**Table 6:** Material consumption analysis of RC structural members according to partial factors in different China’s codes.

$\rho$	Axial tension		Axial compression		Large eccentric compression		Bending		Shear	
	Code^34^	Code^35^	Code^34^	Code^35^	Code^34^	Code^35^	Code^34^	Code^35^	Code^34^	Code^35^
0.1	2.555	2.605	2.448	2.470	2.498	2.532	2.557	2.607	2.431	2.449
0.25	2.920	3.018	2.737	2.768	2.818	2.873	2.923	3.022	2.710	2.735
0.5	3.829	4.048	3.394	3.491	3.621	3.787	3.838	4.057	3.318	3.390
1	5.588	5.932	5.065	5.291	5.392	5.685	5.602	5.947	4.908	5.103
2	8.922	9.483	8.368	8.777	8.754	9.251	8.946	9.508	8.154	8.522

## A case design of RC frame structure

**Figure 5:**
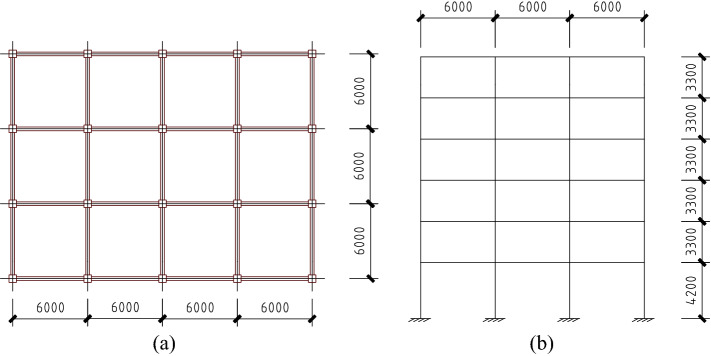
The plan layout and elevation of the RC frame (unit: mm). (**a**) plan layout; (**b**) elevation.

In this section, a case design of RC frame structures is presented to discuss the influence of load partial factors adjustment on reliability, material consumption and safety of RC frame structures in China^53,54^. The plan layout and elevation are shown in Fig. 5. The span and column spacing are 6 m. The total number of floors above the ground is 6, with the bottom floor-to-floor height is 4.2 m and typical floor-to-floor height is 3.3 m. The fortification intensity is VIII (the design acceleration is 0.20 g) and the site classification is II. The characteristic values of dead load and live load of the floors are 5 kN/m^2^ and 2 kN/m^2^ respectively. The characteristic values of dead load of the walls are 8 kN/m^55,56^. The reinforcement HRB400 and HPB300 are used for the longitudinal bar and stirrup respectively. The concrete C30 is used for all columns, beams and slabs. The mechanical properties of steel bars and concrete are described in Table 7.

**Table 7:** Mechanical properties of steel bars and concrete.

Material	Yield strength (Mpa)	Tensile strength (Mpa)	Compressive strength (Mpa)	Elastic modulus (Gpa)
HPB300	300	270	270	210
HRB400	400	360	360	200
C30	–	1.43–	14.3	30

According to the adjustment measures described in the introduction, the frames with different load partial factors are redesigned. Frame 1 is the frame designed according to the load partial factor in China’s old code (GB50153-2008)^34^ and Frame 2 is the frame designed according to new code (GB50068-2018)^35^. In this paper, the models of 6-storey RC frames were carried out in PKPM^57^. The designed sectional dimensions and material information of beams and columns are listed in Table 8.

**Table 8:** Sectional dimensions and material information of beams and columns.

Frame	Sectional dimensions (mm)			Material information		
	Beams	Slabs	Columns	Beams	Slabs	Columns
1	300 × 500	100	600 × 600	C30	C30	C30
2	300 × 600	100	600 × 600			

**Figure 6:**
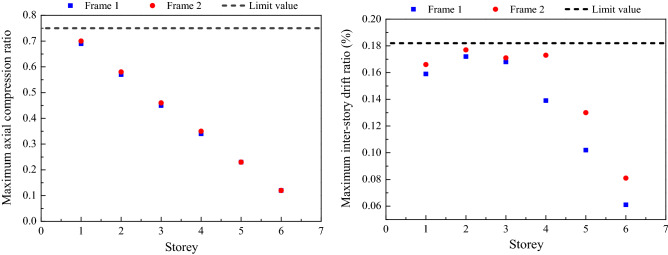
The maximum axial compression ratio and inter-story drift ratio for Frame 1 and Frame 2.

**Figure 7:**
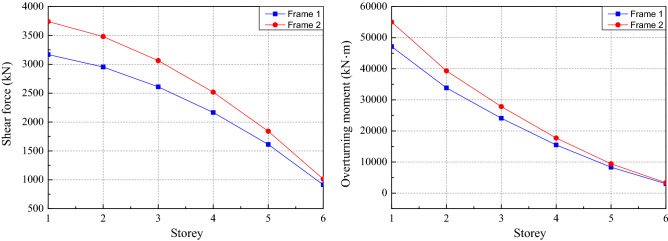
The shear force and the overturning moment for Frame 1 and Frame 2.

**Figure 8:**
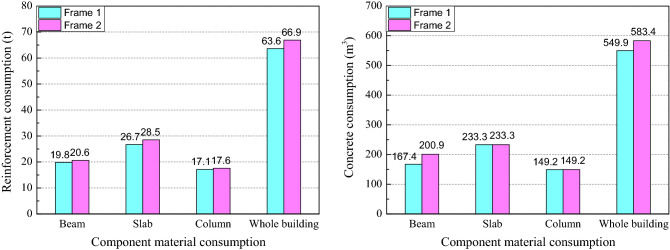
The material consumption of the reinforcement and concrete for Frame 1 and Frame 2.

The maximum axial compression ratio and inter-story drift ratio for Frame 1 and Frame 2 are shown in Fig. 6. As can be seen from Fig. 6, with the designed sectional dimensions and material information of columns are the same for Frame 1 and Frame 2, the maximum axial compression ratios are almost the same. The maximum inter-story drift ratio appears at the second floor. The value of the maximum inter-story drift ratio developed in Frame 2 is 21.4% higher than that of Frame 1 because the adjustment of load partial factors increased the load effect. The shear force and the overturning moment for Frame 1 and Frame 2 are shown in Fig. 7. It can be seen that the maximum of shear force and the overturning moment of Frame 2 are 18.0 and 16.7% higher than that of Frame 1. The material consumption of the reinforcement and concrete for Frame 1 and Frame 2 are shown in Fig. 8. It can be seen that the adjustment of load partial factors mainly result in the increase of reinforcement consumption, while have little effect on the concrete consumption. By comparison, among beam, slab and column, the member with the largest increase in reinforcement (about 6.7%) is the slab.

## Conclusions

The manuscript investigates the reliability design of RC frame structures in China, with special focus on the influence of load partial factors adjustment. A reliability analysis and material consumption analysis of RC structural members designed according to different load partial factor in China’s codes based on First Order Reliability Method are performed. Then, through the PKPM design software, the maximum axial compression ratio, the inter-story drift ratio, the shear force, the overturning moment, the material consumption of the reinforcement and concrete for frames with different load partial factor in China’s codes were studied. The following conclusions are drawn from the present study:

1. Concerning the influence of the load partial factors, the study confirms that, as expected, the reliability increases as the load partial factors improved. The adjustment of load partial factors in design improves the reliability index of RC frame structures and the increase of the reliability index is about 8–16%.
2. The new partial factors in China’s new code are believed to have an increase in the material consumption. The material consumption analysis of RC structural members under the load partial factor in China’s old code and new code shows that the material consumption increases with the increasing of load ratio under all stress statuses and the increase of the material consumption is about 0.75–6.29%.
3. For the specifically configured Frame 1 and Frame 2 considered in this paper, the maximum axial compression ratio, the inter-story drift ratio, the shear force, the overturning moment, the material consumption of the reinforcement and concrete with different load partial factor in China’s codes were studied. The maximum axial compression ratios are almost the same for Frame 1 and Frame 2. The maximum inter-story drift ratio developed in Frame 2 is 21.4% higher than that of Frame 1. The maximum of shear force and the overturning moment of Frame 2 are 18.0 and 16.7% higher than that of Frame 1. The adjustment of load partial factors mainly result in the increase of reinforcement consumption, while have little effect on the concrete consumption. And the member with the largest increase in reinforcement is the slab.

This study provides an analytical and conclusive insight into the influence of load partial factor adjustment on safety level and material consumption of RC frame structures. However, this paper only considers the simple load combinations and RC frame structures. Further studies are being developed on the seismic action combinations and other structural component forms, based on the promising achievements in this work. Also, since reliability analysis is highly influenced by the statistical parameters of for loads and resistance, new values of statistical parameters covering historical data is needed to obtain more accurate results.

## Data Availability

The datasets used and/or analyzed during the current study available from the corresponding author on reasonable request.
